# Comparison of Single and Repeated Dosing of Anti-Inflammatory Human Umbilical Cord Mesenchymal Stromal Cells in a Mouse Model of Polymicrobial Sepsis

**DOI:** 10.1007/s12015-021-10323-7

**Published:** 2022-01-10

**Authors:** Barbara Fazekas, Senthilkumar Alagesan, Luke Watson, Olivia Ng, Callum M. Conroy, Cristina Català, Maria Velascode Andres, Neema Negi, Jared Q. Gerlach, Sean O. Hynes, Francisco Lozano, Stephen J. Elliman, Matthew D. Griffin

**Affiliations:** 1 Regenerative Medicine Institute at CÚRAM Centre for Research in Medical Devices, School of Medicine, National University of Ireland Galway, Galway, Ireland; 2 Orbsen Therapeutics Ltd., Galway, Ireland; 3 Institut d’Investigacions Biomèdiques August Pi i Sunyer, Barcelona, Spain; 4 Glycoscience Group, National Centre for Biomedical Engineering Science, National University of Ireland Galway, Galway, Ireland; 5 Discipline of Pathology, School of Medicine, National University of Ireland Galway, Galway, Ireland; 6 Department of Histopathology, Galway University Hospitals, Galway, Ireland; 7 Servei d’Immunologia, Hospital Clínic de Barcelona, Barcelona, Spain; 8 Department de Biomedicina, Universitat de Barcelona, Barcelona, Spain; 9 Department of Nephrology, Saolta University Health Care Group, Galway University Hospitals, Galway, Ireland; 10 National University of Ireland Galway, REMEDI, Biomedical Sciences, Corrib Village, Dangan, Galway, H91 TK33 Ireland

**Keywords:** Acute kidney injury, Mesenchymal stromal cell, Regenerative medicine, Sepsis, Inflammation, Cell therapy

## Abstract

**Summary:**

Mesenchymal stromal cells (MSCs) ameliorate pre-clinical sepsis and sepsis-associated acute kidney injury (SA-AKI) but clinical trials of single-dose MSCs have not indicated robust efficacy. This study investigated immunomodulatory effects of a novel MSC product (CD362-selected human umbilical cord-derived MSCs [hUC-MSCs]) in mouse endotoxemia and polymicrobial sepsis models. Initially, mice received intra-peritoneal (i.p.) lipopolysaccharide (LPS) followed by single i.p. doses of hUC-MSCs or vehicle. Next, mice underwent cecal ligation and puncture (CLP) followed by intravenous (i.v.) doses of hUC-MSCs at 4 h or 4 and 28 h. Analyses included serum/plasma assays of biochemical indices, inflammatory mediators and the AKI biomarker NGAL; multi-color flow cytometry of peritoneal macrophages (LPS) and intra-renal immune cell subpopulations (CLP) and histology/immunohistochemistry of kidney (CLP). At 72 h post-LPS injections, hUC-MSCs reduced serum inflammatory mediators and peritoneal macrophage M1/M2 ratio. Repeated, but not single, hUC-MSC doses administered at 48 h post-CLP resulted in lower serum concentrations of inflammatory mediators, lower plasma NGAL and reversal of sepsis-associated depletion of intra-renal T cell and myeloid cell subpopulations. Hierarchical clustering analysis of all 48-h serum/plasma analytes demonstrated partial co-clustering of repeated-dose hUC-MSC CLP animals with a Sham group but did not reveal a distinct signature of response to therapy. It was concluded that repeated doses of CD362-selected hUC-MSCs are required to modulate systemic and local immune/inflammatory events in polymicrobial sepsis and SA-AKI. Inter-individual variability and lack of effect of single dose MSC administration in the CLP model are consistent with observations to date from early-phase clinical trials.

**Graphical Abstract:**

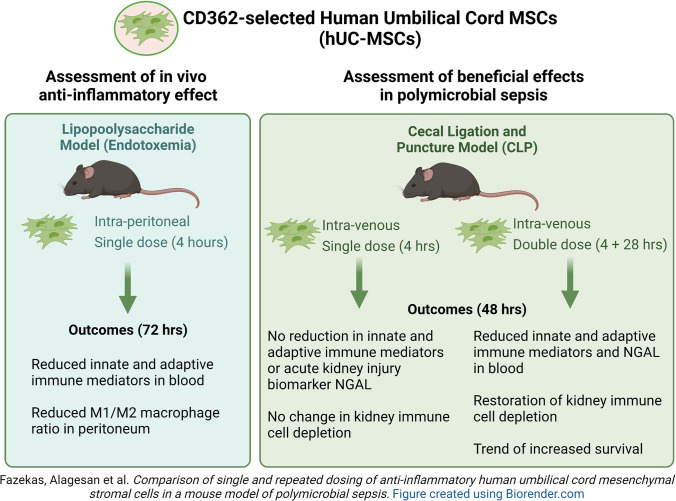

**Supplementary Information:**

The online version contains supplementary material available at 10.1007/s12015-021-10323-7.

## Introduction

Sepsis is characterised by life-threatening organ dysfunction due to a dysregulated host response to infection caused by bacterial, fungal, viral, and parasitic pathogens [1]. Each year, sepsis affects nearly 50 million people worldwide, frequently leading to severe systemic consequences and organ injuries, including acute kidney injury (AKI), resulting in approximately 11 million deaths [2, 3]. Mesenchymal stromal cells (MSC) are multipotent cells with extensive immune-modulatory properties that can be isolated from various tissues [4]. It is now well recognised that MSC offer a potential disease-modulating therapy for sepsis and sepsis-associated AKI (SA-AKI) [5, 6]. Several recent studies using mouse and rat cecal ligation and puncture (CLP), lipopolysaccharide (LPS) or fecal peritonitis models have shown that systemic administration of MSC has the potential to reduce inflammation, counteract bacterial infection and improve the repair of injured tissue in sepsis [7–11], including SA-AKI, [10, 12–15] by modulating the balance between pro-inflammatory and anti-inflammatory states.

A recent trial in healthy adults confirmed that preventative intravenous (i.v.) treatment with 4 × 10^6^ MSC/kg produces early immunomodulatory effects on the host response to LPS [16]. Furthermore, administration of allogeneic MSC to patients with septic shock (NCT01849237, NCT02421484, and NCT02328612) and sepsis-related acute respiratory distress syndrome (NCT01775774) demonstrated good safety and tolerability in Phase 1 clinical trials [16–19]. Despite these encouraging results, Galstyan et al. also reported that a single dose of i.v. MSC did not prevent death from sepsis-related organ dysfunction, raising the possibility that additional doses may be necessary to derive meaningful clinical benefit [17]. A recent non-sepsis, phase 2 trial (NCT01602328) in which single doses of allogeneic MSC were delivered intra-aortically to patients with sterile AKI following cardiopulmonary by-pass surgery also failed to demonstrate a beneficial effect on organ dysfunction and patient survival despite promising pre-clinical and phase 1 trial results [20]. Whether repeated dosing during the early course of sepsis and other acute inflammatory syndromes could augment or extend the disease-modulating effects of MSC remains relatively under-investigated at pre-clinical and translational levels. Furthermore, incomplete knowledge of the mechanism of action, dose response and optimal clinical indices for MSC administration in sepsis limits the potential for designing successful trials [5, 21, 22].

In the current study, we performed pre-clinical investigation of the anti-inflammatory effects of CD362-selected human umbilical cord-derived MSC (hUC-MSC) in mouse models of sepsis. This surface marker-selected hUC-MSC is a novel therapeutic product that has demonstrated evidence of efficacy in rat models of bacterial pneumonia and sepsis when administered early after disease onset [23, 24]. As a clinical-grade investigational medicinal product (IMP), hUC-MSC is currently undergoing phase I/II trial in patients with moderate to severe acute respiratory distress syndrome (ARDS) due to COVID-19 (NCT03042143) and in multiple autoimmune inflammatory diseases (POLARISE). We aimed to demonstrate the anti-inflammatory potential of single doses of hUC-MSC in mouse models of LPS- and CLP-induced endotoxemia/polymicrobial sepsis [25, 26] and, in the latter, to determine whether a second administration of hUC-MSC during the early disease course resulted in greater or more frequent beneficial effects on systemic inflammation and organ-specific injury, exemplified by SA-AKI.

## Materials & Methods

### Cells

Anti-CD362^+^-selected hUC-MSC were cultured from ethically-sourced human umbilical cord tissue obtained from Tissue solutions Ltd. (Glasgow, U.K). Primary isolation and expansion cultures of CD362^+^ hUC-MSC was carried out as previously described [19, 23, 27]. Cryopreserved vials (1 × 10^7^ in 1 mL) of anti-CD362^+^-selected hUC-MSC were thawed, transferred into 9 mL of phosphate buffered saline (PBS). After live/dead analysis either via trypan blue dye or automated cell counter (NucleoCounter® NC-200™, Chemometec A/S, Denmark), the required numbers of cells were pelleted via centrifugation at 400×g for 5 min and the cells were re-suspended in 100 μl of sterile saline for intra-peritoneal (i.p.) or i.v. injection.

### Animal Procedures

All animal procedures were carried out under a license (no. 255/17) from the Animal Experimentation Ethical Committee, University of Barcelona and under authorisation (AE19125/P082 and AE19125/P066) from the Health Products Regulatory Authority, Ireland, and approved by the NUI Galway Animal Care Research Ethics Committee. All procedures were performed in licensed animal facilities at NUI Galway and University of Barcelona.

For the LPS model of endotoxemia, 10-12 week-old male C57BL/6 mice from Charles River Ltd., Kent, UK were used. Mice were injected i.p. with 5 μg/g LPS (LPS 0111:B4, catalogue no. L2630, Sigma Aldrich, UK) in 100 μl of sterile saline. The mice were housed in groups of 3-5 mice/cage during the study in individually ventilated cages. Single i.p. injections of 2.5 × 10^5^ hUC-MSC or equal volumes of vehicle (sterile saline) were administered 4 h after LPS injections. The animals were monitored every 4 h until the end of the study using a distress score sheet and support measures according to a pre-determined protocol. Humane euthanasia was performed at the defined experimental end-point or earlier if animals exceeded the pre-defined severity score threshold. At the time of euthanasia, peritoneal exudates were collected for flow cytometry analysis by carefully flushing 5 mL of sterile PBS into and out of the peritoneal cavity.

Cecal ligation and puncture (CLP) was performed on 8-12 week-old, male C57BL/6 mice (Charles River Ltd., UK). The mice received buprenorphine 0.1 mg/kg (Richter Pharma AG, Austria) subcutaneously 25-30 min before the procedure and were anesthetized with 1.8-2% isoflurane (with O_2_ flow of 0.5 L/min) at NUI Galway or with Anesketin (100 mg/mL; Dechra Veterinary Products SLU, Spain) and Rampun (20 mg/mL; Bayer, Germany) at University of Barcelona. The lower half of the abdomen was shaved and cleaned with 4% chlorhexidine or povidone-Iodine and incised 1 cm vertically along the midline. The cecum was externalized and the distal 50% was ligated using 4.0-6.0 M sutures. Cecal material was released by ‘through and through’ puncture with a 21-gauge needle and a drop of fecal matter was exuded before reinstating the cecum into the peritoneal cavity and suturing the muscle and skin closed. Sham-operated mice underwent an identical procedure, including opening the peritoneum and exposing the bowel, but without ligation and perforation of the cecum. Mice received 0.5 mL of Gelofusine (Braun Melsungen AG, Germany) by i.p. instillation prior to wound closure. Post-operative support consisted of buprenorphine diluted given subcutaneously (s.c.) every 8-12 h until the pre-determined end-points (48 or 72 h for individual experiments). Administration of 1 × 10^6^ hUC-MSC or equivalent volumes of vehicle (sterile saline) was carried out i.v. via the tail vein at 4 h or at 4 and 28 h following CLP. Frequent monitoring and support was carried out according to an ethically-approved protocol. Humane euthanasia was performed at the defined experimental end-point or earlier if animals exceeded a pre-defined severity score threshold.

The cell doses for the two animal models employed for the study were selected on the basis of prior reports of human MSC anti-inflammatory effects in similar models [9, 13, 14, 28, 29]. The group sizes for the LPS study were selected empirically based on relevant prior reports for this model [30, 31]. The group sizes for the CLP study were determined for a primary outcome of plasma NGAL at 24 h post-surgery. Using data from a pilot experiment, a sample size of 9 animals per group was calculated to provide 90% power assuming a 5% significance level and a two-sided test (http://www.3rs-reduction.co.uk/html/6__power_and_sample_size.html). An expected attrition rate of 10% was applied to select the final group size of *n* = 10.

### Blood Sampling and Tissue Procurement

Venous blood samples to a maximum volume of 20 μL were drawn intermittently from tail and facial veins by aseptic technique using 25-21 gauge needles and were collected into heparin (VWR International, Dublin, Ireland)-containing tubes. A terminal blood sample was drawn by cardiac puncture at the time of euthanasia. Serum was collected in micro-tubes with serum gel and clotting activator (Sarstedt, Wexford, Ireland). Plasma and serum samples were prepared by centrifugation at 10,000×g for 10 min. Serum samples were stored at −80 °C and subsequently analysed for biochemical parameters by NationWide Laboratories (Lancashire, UK). Spleen, lungs, kidneys and liver were dissected immediately after euthanasia.

### Multicolour Flow Cytometry

For flow cytometry of mouse peritoneal macrophages (LPS model), 100 μL of freshly-prepared peritoneal exudate cells re-suspended in FACS buffer (PBS, 2% fetal calf serum and 0.05% sodium azide) were labelled with the following combinations of fluorochrome-labelled antibodies at 4 °C for 30 min: anti-Ly6C-PerCP-Cy™5.5 (clone-AL-21), anti-CD11b-APC-Cy7 (clone-M1/70), anti-CD68-PE (F1/11), anti-CD206-APC (MR5D3) from BD Pharmingen™ (BD Bioscience, Berkshire, UK). Cell viability was analysed by SYTOX™ blue dead cell stain (Thermo-Fisher Scientific, Dublin, Ireland) according to manufacturer’s instructions. For flow cytometry of kidney cells, single-cell suspensions were prepared from freshly-dissected kidneys by collagenase/DNase digestion and were enriched for CD45^+^ bone marrow-derived cells by magnetic column separation. Single-cell suspensions of lung were also prepared by collagenase/DNase digestion and mechanical disruption respectively without subsequent CD45-enrichment. Single cell suspensions of spleen were prepared by mechanical disruption. Kidney cell suspensions were stained with panels of fluorochrome-coupled monoclonal antibodies. A detailed protocol for preparation and flow cytometry analysis of cell suspensions is available in Supplementary Methods. For all flow cytometry analyses, labelled cells were washed and re-suspended in FACS buffer and immediately analysed on a FACS Canto II cytometer (BD Biosciences). Data files were subsequently analyzed using FlowJo v6 software (Ashland, OR, USA). Details and examples of the gating strategies used to define and enumerate specific immune cell subpopulations are provided in Supplementary Table 1 and Supplementary Fig. 1.

### Immunoassays

Plasma neutrophil gelatinase-associated lipocalin (NGAL) concentration was quantified with the mouse Lipocalin-2/NGAL Duo-Set ELISA Development kit (R&D Systems, Minneapolis, MI, USA) according to the manufacturer’s suggested protocol (details in Supplementary Methods). For multiplex quantification of cytokines and chemokines in serum, the Bio-Plex Pro mouse cytokine standard 23-plex assay (Bio-Rad, Accuscience) was used according the manufacturer’s instructions. Samples were analysed on a Bioplex 200 multiplex ELISA system (Bio-Rad, Accuscience).

### Histology and Immunohistochemistry

Kidneys were dissected and placed in 10% neutral buffered formalin for 24 h at room temperature before being processed in a Leica Tissue Processor ASP300. Tissues were wax-embedded in a Leica EG1150 wax embedder fitted with EG1130 Cold Plate, and 5-μm sections were cut using a Leica RM12235 microtome. Sections were transferred to Superfrost Plus microscope slides (Fisher Scientific Ireland) and dried overnight at room temperature. Histologic staining of sections for hematoxylin and eosin (H&E) and periodic acid-Schiff (PAS) were performed using standard protocols (details provided in Supplementary Methods).

For immunohistochemical staining, kidney tissue sections were dewaxed in xylene then hydrated in graded ethanol solutions. Heat-mediated antigen retrieval was performed with Tris/EDTA buffer pH 9.0 at 90 °C for 30 min. Sections were treated with 0.3% H_2_O_2_, and incubated with avidin-biotin blocking solution (SP-2001, Vector Laboratories, Burlingame, CA, USA) to reduce nonspecific staining. Next, the slides were incubated for one hour at 4 °C with rabbit anti-mouse NGAL monoclonal antibody (1:2000; ab216462, Abcam, Cambridge, UK), followed by incubation with biotinylated goat anti-rabbit IgG secondary antibody (BA-1000). For colorization, an avidin-biotin horseradish peroxidase complex (Vector Laboratories) and 3,3-diaminobenzidine substrate solution (Sigma-Aldrich) were applied to the slides at room temperature for 30 and 5 min respectively and the slides were counterstained with Gill no. 3 hematoxylin (Sigma-Aldrich) for 30 s. Negative control slides were prepared by staining under identical conditions but without adding the primary antibody.

### Semi-Quantitative Scoring of Kidney Tissue Sections

Stained sections of kidney were analyzed in blinded fashion by light microscopy at 40X magnification using an Olympus BX43 bright-field microscope (Olympus, Center Valley, PA) and with IS TCapture software (Tucsen Photonics Co., Fujian, China). For each kidney, twenty non-overlapping fields of a stained section were captured, and the positively stained area was scored by a blinded observer for (A) tubular dilatation, cast and necrosis (PAS) and (B) NGAL expression [32] Scoring was carried out on a 0-4 semi-quantitative scoring scale (details in **Supplementary Methods**). Mean scores were calculated for each individual kidney, and final results were expressed as group means ± SD.

### Statistical Analysis

Results were expressed as means ± SD, and differences between conditions were tested statistically by ANOVA and post-hoc tests where indicated using GraphPad Prism 5 software (GraphPad Software, La Jolla, CA). Significance was assigned at *p* < 0.05. Unsupervised hierarchical clustering and the generation of the corresponding heat map were performed using the Morpheus online visualization and analysis suite (Broad Institute, Cambridge, MA, https://software.broadinstitute.org/morpheus). All data, except gamma glutamyl-transpeptidase (Gamma GT) and albumin-globulin ratio (Albumin:Globulin), were subjected to log2 transformation in Excel (version 2013, Microsoft, Redmond, WA), prior to clustering. The hierarchical relationship of data patterns for all animals was establish by average linkage, Euclidean distance method.

## Results

### A Single Dose of hUC-MSC Modulated Inflammatory Response to LPS in Mice

**Fig. 1:**
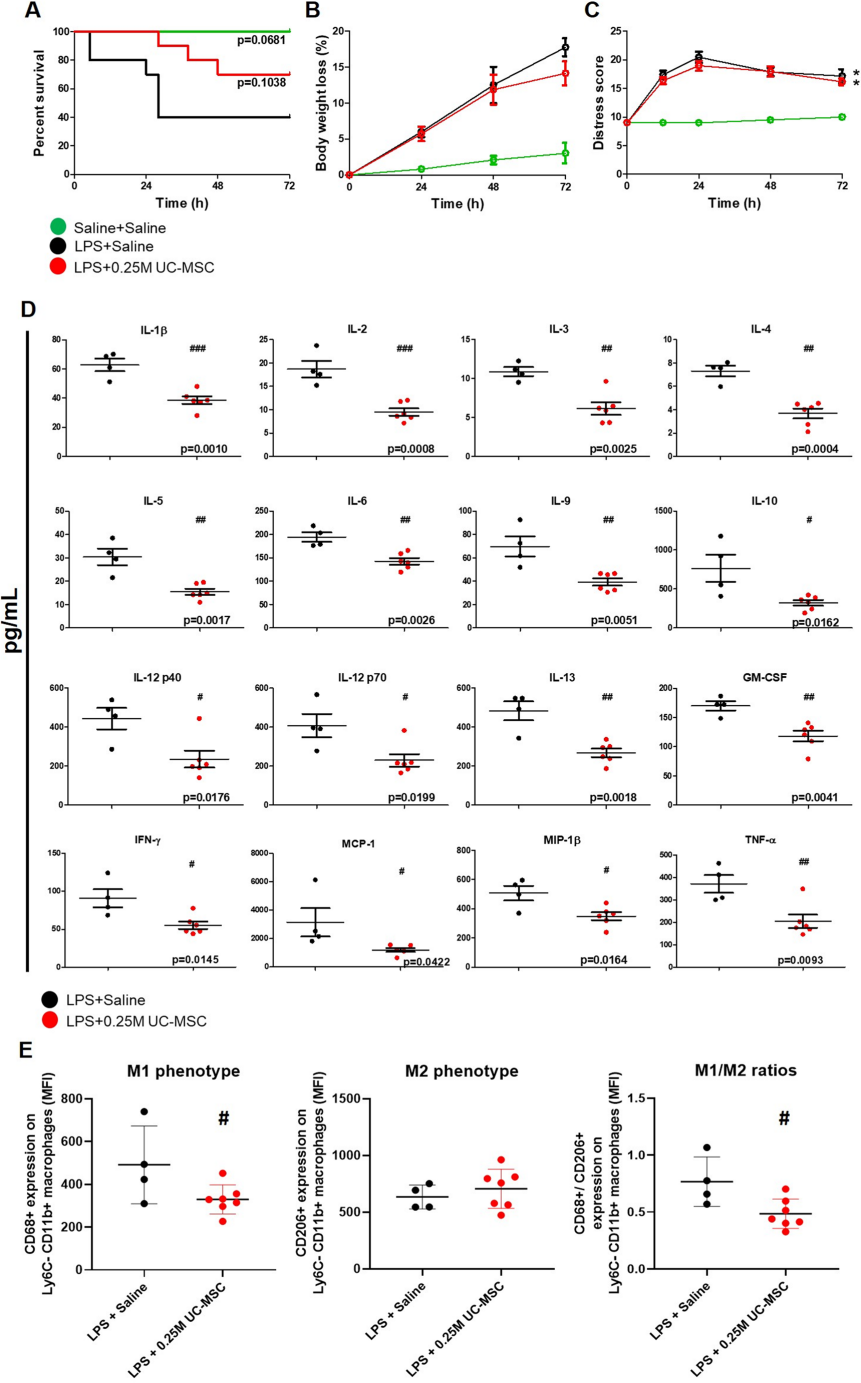
*Effects of single-dose intra-peritoneal hUC-MSC administration on survival, systemic inflammatory response and peritoneal macrophage polarization following LPS:* **a**-**c** Survival curve, percentage of body weight loss data and distress score of groups of mice over 72 h following intra-peritoneal injection of saline alone (SHAM+Saline [*n* = 4]), LPS and saline (LPS + Saline [*n* = 10]) or LPS and 0.25 × 10^6^ hUC-MSC (LPS + 0.25 M UC-MSC [*n* = 10]). **d** Serum concentrations of inflammatory cytokines and chemokines in mice surviving to 72 h following LPS and saline (LPS + Saline [*n* = 5]) or LPS and 0.25 × 10^6^ hUC-MSC (LPS + 0.25 M UC-MSC [*n* = 5]). **e** Mean fluorescence intensities (MFI) of Ly6C^−^/CD11b^+^ peritoneal macrophages for M1 marker CD68 (left) and M2 marker CD206 (middle) as well as CD68/CD206 ratios for mice surviving to 72 h following LPS and saline (LPS + Saline [*n* = 4]) or LPS and 0.25 × 10^6^ hUC-MSC (LPS + 0.25 M UC-MSC [*n* = 5]). Statistical analysis: A) Long-rank test of Kaplan-Meier survival curve, *p* values indicate statistical comparison with LPS + Saline group. B) Repeated measures ANOVA with Bonferroni post-test. C) Friedman test with Dunn’s post-test, 95% confidence interval. D) Non-Gaussian distribution: Unpaired T test, *p* < 0.05. Gaussian distribution: Non-parametric Mann-Whitney test *p* < 0.05. E) Unpaired t-test. *Significantly different from SHAM groups (*p* < 0.05). ^#^Significantly different from LPS + Saline group (^#^*p* < 0.05, ^##^*p* < 0.01, ^###^*p* < 0.001)

To initially establish a modulatory effect of hUC-MSC on acute systemic inflammatory response in mice, the LPS endotoxemia model was used. As shown in Fig. 1a, i.p. administration of 2.5 × 10^5^ hUC-MSC 4 h after LPS injection was associated with higher 72-h survival compared to a control group that received LPS followed by i.p. saline (70% vs. 40% survival). However, the difference did not reach statistical significance and the average body weight losses and total distress scores over 72 h were closely comparable for the two groups (Fig. 1b and c). Despite the limited effects on physical determinants of acute illness, multiplexed analysis of soluble inflammatory mediators in serum of animals that survived to 72 h revealed significant reductions in hUC-MSC recipients in comparison to the saline group (Fig. 1d). Notably, circulating concentrations of key mediators of innate (e.g. IL-1β, IL-6, GM-CSF, TNFα, IL-10, MCP-1, MIP-1β) and Th1- and Th2-type adaptive immune responses (e.g. IL-2, IFNγ, IL-12, IL-3, IL-4, IL-5, IL-13, IL-9) were lower in hUC-MSC-treated compared to saline-treated animals. Concentrations of the Th17-type mediator IL-17, the eosinophil chemoattractant, eotaxin and the chemokine CCL5 were unaffected by hUC-MSC administration (data not shown). To assess the immunomodulatory effects of hUC-MSC at the site of delivery, CD11b^+^/Ly6C^−^ peritoneal macrophages from surviving animals were analysed by flow cytometry for surface expression levels of the M1 and M2 markers CD68 and CD206. As shown in Fig. 1e, peritoneal macrophages from hUC-MSC-treated animals had lower CD68 surface expression and lower CD68/CD206 ratio compared to those from saline-treated animals. Overall, these results demonstrated that a single dose of hUC-MSC had distinct anti-inflammatory and immune-modulatory effects in a simplified sepsis-like model with limited influence on overall disease severity.

### Repeated Doses of hUC-MSC Modified Survival and Systemic Inflammation Following CLP-Induced Polymicrobial Sepsis in Mice

**Fig. 2:**
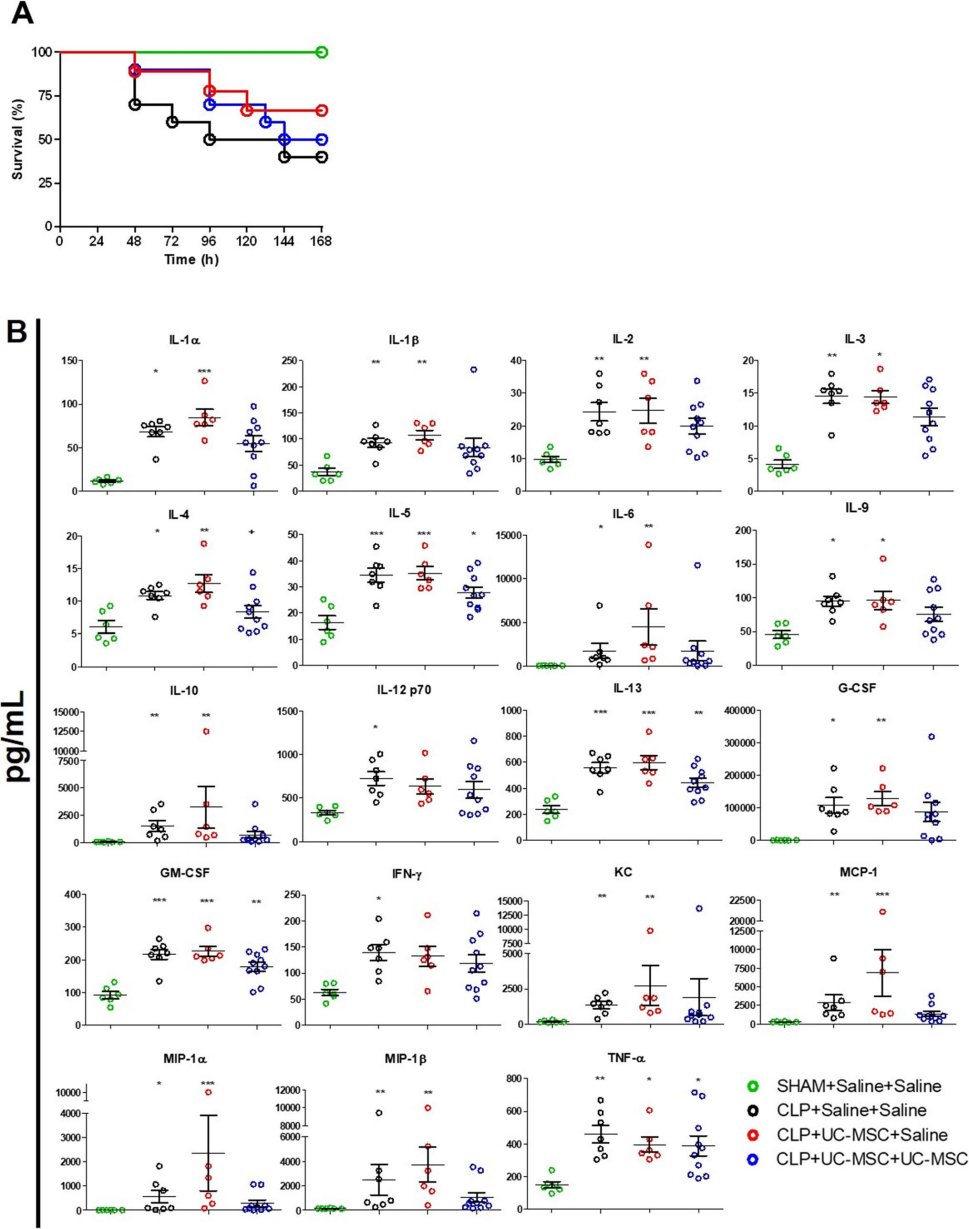
*Effects of single- and double-dose intravenous hUC-MSC on survival and systemic inflammatory responses following CLP-induced polymicrobial sepsis:* **a** Survival curves for groups of mice following sham procedure with saline injections at 4 and 28 h (SHAM+Saline+Saline, [*n* = 5]), CLP procedure with saline injections at 4 and 28 h (CLP + Saline+Saline, [*n* = 10]), CLP procedure with 1 × 10^6^ hUC-MSC at 4 h and saline injection at 28 h (CLP + UC-MSC + Saline, [*n* = 9]) or CLP procedure with 1 × 10^6^ hUC-MSC at 4 h and at 28 h (CLP + UC-MSC + UC-MSC, [*n* = 9]). Animals were monitored for 168 h following procedures. **b** Serum concentrations of inflammatory cytokines and chemokines at 48 h following the same combinations of procedures and treatments. Groups sizes for the multiplex assay analyses were SHAM+Saline+Saline (*n* = 6), CLP + Saline+Saline (*n* = 7) and CLP + UC-MSC + Saline (*n* = 6) or CLP + UC-MSC + UC-MSC (*n* = 10). Statistical analysis: **a** Long-rank test of Kaplan Meier survival curve **b** Non-Gaussian distribution: Kruskal-Wallis test with Dunn’s multiple comparison test, *p* < 0.05. Gaussian distribution: One-way ANOVA with Bonferroni multiple comparison post-test and with 95% confidence interval. *Significantly different from SHAM group (*p < 0.05, **p < 0.01, ***p < 0.001)

To assess (i) whether the anti-inflammatory effects of hUC-MSC could be reproduced in a more clinically-relevant model and route of administration and (ii) whether a repeat dose of cells early in the disease course could more potently modulate the course of sepsis, experiments were subsequently performed in the mouse CLP model. Groups of animals underwent CLP or sham procedures and received i.v. injections of 1 × 10^6^ hUC-MSC or equivalent volumes of saline at 4 h only or at 4 and 28 h after CLP. In the first such experiment, survival over a prolonged period (7 days) was determined (Fig. 2a). As shown, CLP animals administered saline alone at both time points had 40% 7-day survival compared to 100% for sham controls. Seven-day survival of groups that received hUC-MSC at 4 h only (single dose) or at 4 and 28 h (double dose) did not differ significantly from that of the untreated group. It was noted, however, that mortality of animals in both hUC-MSC groups tended to occur later than that of untreated CLP animals. To explore this further, a second study with a duration of 48 h was performed using the same groupings and group sizes. As shown in Table 1, when the 48-h survivals of the combined total animals for each of the 4 groups from the 2 experiments were compared, those that received two doses of hUC-MSC had 95% survival compared to 75% and 74% respectively for untreated and single dose-treated groups. This difference in survival did not reach statistical significance.

**Table 1:** Survival to 48 h following CLP in combined groups from two experiments comparing effects of saline, single-dose hUC-MSC and double-dose hUC-MSC

	Time-point (No. of animals surviving)					
Experimental Group (Procedure/IV inject. at 4 + 28 h)	0 h	12 h	24 h	36 h	48 h	% Survival to 48 h
SHAM / Saline + Saline	14	14	14	14	14	100
CLP / Saline + Saline	20	20	20	20	15	75
CLP / hUC-MSC + Saline (single-dose)	19	19	19	19	14	74
CLP / hUC-MSC + hUC-MSC (Double-dose)	20	20	20	20	19	95

To investigate further, serum samples collected from surviving animals at the end-point (48 h post-CLP) of the second experiment were analyzed by multiplex assay for soluble mediators of innate and adaptive immune responses (Fig. 2b). As shown, serum concentrations of the majority of these cytokines and chemokines were significantly elevated in untreated CLP compared to sham animals. As with the LPS model, serum concentrations of IL-17, eotaxin and CCL5 were unaffected by CLP (data not shown). With the exception of IL-12p70 and IFNγ, all CLP-induced mediators were also significantly elevated in the single-dose hUC-MSC-treated group. For the double-dose hUC-MSC group, however, multiple innate immune mediators (IL-1α, IL-1β, IL-6, IL-10, G-CSF, KC, MCP-1, MIP-1α, MIP-1β) and some Th1- and Th2-type adaptive immune mediators (IFNγ, IL-2, IL-3, IL-9) did not differ significantly from the sham group. The findings supported a conclusion that repeated dosing of hUC-MSC is necessary for detectable modulation of systemic inflammation 48 h after onset of polymicrobial sepsis.

### The CLP Model Was Not Associated with Multi-Organ Failure

**Fig. 3:**
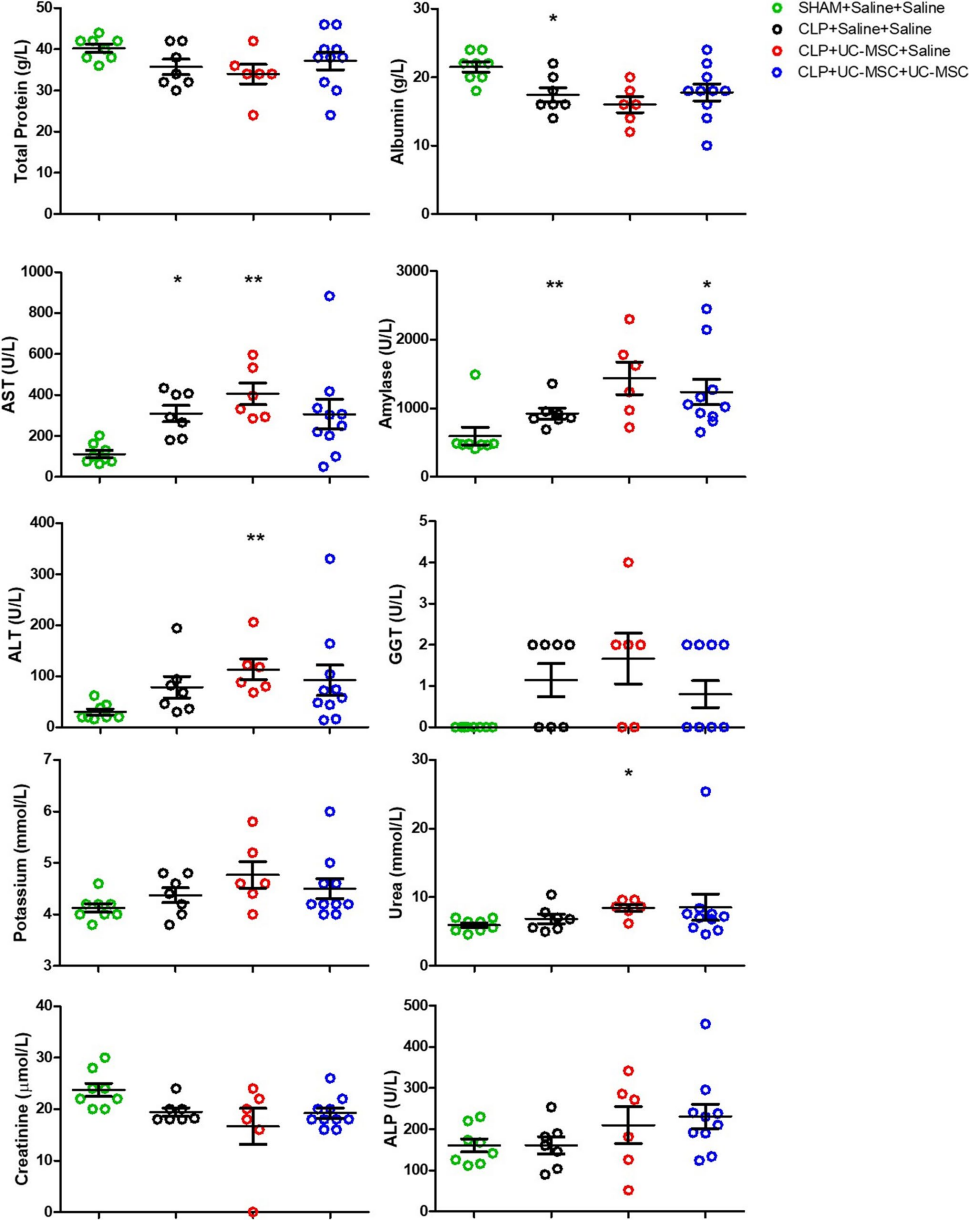
*Results of serum biochemistry assays at 48 h following Sham procedure or CLP procedure with injections of saline or hUC-MSC:* Results for serum biochemical assays at 48 h post-procedure are shown for the following groups: sham procedure with saline injections at 4 and 28 h (SHAM+Saline+Saline, [*n* = 8]), CLP procedure with saline injections at 4 and 28 h (CLP + Saline+Saline, [*n* = 7]), CLP procedure with 1 × 10^6^ hUC-MSC at 4 h and saline injection at 28 h (CLP + UC-MSC + Saline, [*n* = 6]) or CLP procedure with 1 × 10^6^ hUC-MSC at 4 h and at 28 h (CLP + UC-MSC + UC-MSC, [*n* = 10]). Statistical analysis: Non-Gaussian distribution: Kruskal-Wallis test with Dunn’s multiple comparison test, *p* < 0.05. Gaussian distribution: One-way ANOVA with Bonferroni multiple comparison post-test and with 95% confidence interval. *Significantly different from SHAM group (*p < 0.05, **p < 0.01). Abbreviations: AST = Aspartate Aminotransferase; ALT = Alanine Aminotransferase; GGT = Gamma-Glutamyl Transferase; ALP = Alkaline Phosphatase

To determine whether the CLP model was associated with multi-organ failure that may have been modulated by single- and/or double-dose hUC-MSC administration, biochemical analyses reflecting liver, kidney and pancreas function were performed on 48-h serum samples collected at the termination of the second experiment (Fig. 3). As shown, there were trends for individual group results consistent with adverse changes in liver and pancreas function tests (AST, albumin, Amylase, ALT, GGT) in CLP compared to sham groups. In contrast, apart from a mild increase in urea in one group, serum markers of kidney function (creatinine, urea, potassium) were not different among the groups. These results indicated that, while the CLP model we established resulted in systemic inflammation and reduced survival, multi-organ failure was not present at the time and anti-inflammatory effects of repeated hUC-MSC administration were observed.

### Repeated Dosing of hUC-MSC Modulated the Severity of Acute Kidney Injury Following CLP-Induced Polymicrobial Sepsis in Mice

**Fig. 4:**
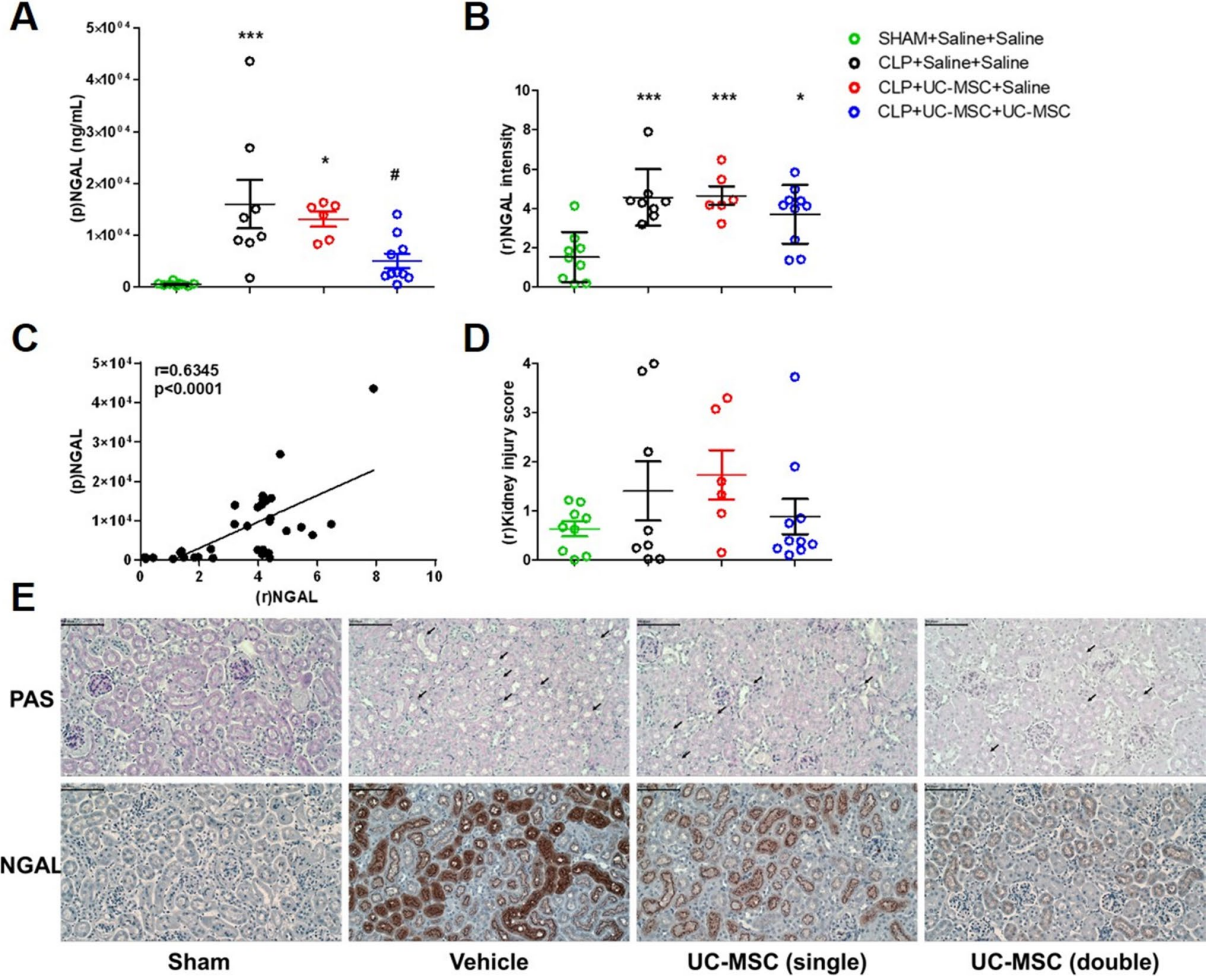
*Markers of acute kidney injury 48 h following Sham or CLP procedures with saline, single-dose hUC-MSC and double-dose hUC-MSC:* **a** Plasma (p)NGAL concentrations. **b** Semi-quantitative scoring of renal tubular (r)NGAL staining intensity in kidney sections **c** Scatter plot illustrating the correlation between 48-h (p)NGAL and (r)NGAL for individual animals from all CLP groups. **d** Total kidney injury scores, representing the sum of individual semi-quantitative scores for tubular dilatation, cast formation and necrosis in PAS-stained 48-h kidney tissue sections. **e** Representative examples of PAS and NGAL staining of 48-h kidney sections. Arrows show tubular dilatation, cast and necrosis. NGAL expression showed peritubular appearance in the cortex of the kidney. Magnification 10X. Bar = 100 μm. Group sizes: SHAM+Saline+Saline (*n* = 9), CLP + Saline+Saline (*n* = 8), CLP + UC-MSC + Saline (*n* = 6) and CLP + UC-MSC + UC-MSC (*n* = 10). Statistical analysis: (A-B) One-way ANOVA with Bonferroni multiple comparison post-test and with 95% confidence interval (C) Two-tailed Spearman correlative testing with regression line (D) Kruskal-Wallis test, Dunn’s multiple comparison test, *p* < 0.05. *Significantly different from SHAM group (*p < 0.05, **p < 0.01, ***p < 0.001). #Significantly different from CLP group (*p* < 0.05)

To more clearly determine whether the anti-inflammatory effect of repeated hUC-MSC administration was associated with end-organ protection, we focussed on the kidneys. Additional analyses of AKI were performed on 48-h samples from each group of the second CLP experiment. Neutrophil gelatinase-associated lipocalin-2 is a biomarker that increases rapidly in blood and urine following the onset of sterile AKI as well as SA-AKI [33, 34]. As shown in Fig. 4a, plasma NGAL concentrations were markedly increased in CLP compared to sham control animals at 48 h. In the double-, but not the single-dose, hUC-MSC group, however, the average plasma NGAL of animals surviving to 48 h was lower than that of the untreated CLP group. At the same time-point, the intensity of renal tubular staining for NGAL, determined by immunohistochemical staining of kidney tissue sections, was also markedly increased in CLP compared to sham controls (Fig. 4b and e). Although not reaching statistical significance, there was a trend towards lower renal tubular NGAL staining among the double-dose hUC-MSC group compared to untreated and single-dose hUC-MSC groups. To support the conclusion that plasma NGAL reflected severity of AKI rather than being a non-specific marker of sepsis-associated inflammation, there was a significant correlation between plasma NGAL and renal tubular NGAL staining intensity at 48 h among all CLP animals (Fig. 4c). To determine whether overt tubular necrosis and/or inflammatory renal injury was present, semi-quantitative scoring of PAS-stained tissue sections for tubular damage and interstitial cell infiltrates was carried out. This analysis failed to demonstrate clear differences among the sham and CLP groups (Fig. 4d and e). Furthermore, a blinded analysis carried out by a consultant histopathologist (SOH) for features of ischemia (necrosed tubules, loss of PAS^+^ brush border, cell swelling and oedema, the presence of protein casts, neutrophils and capillary collapse) indicated only mild, variable abnormalities that did not differ between Sham and CLP groups or among the three CLP groups (data not shown).

**Fig. 5:**
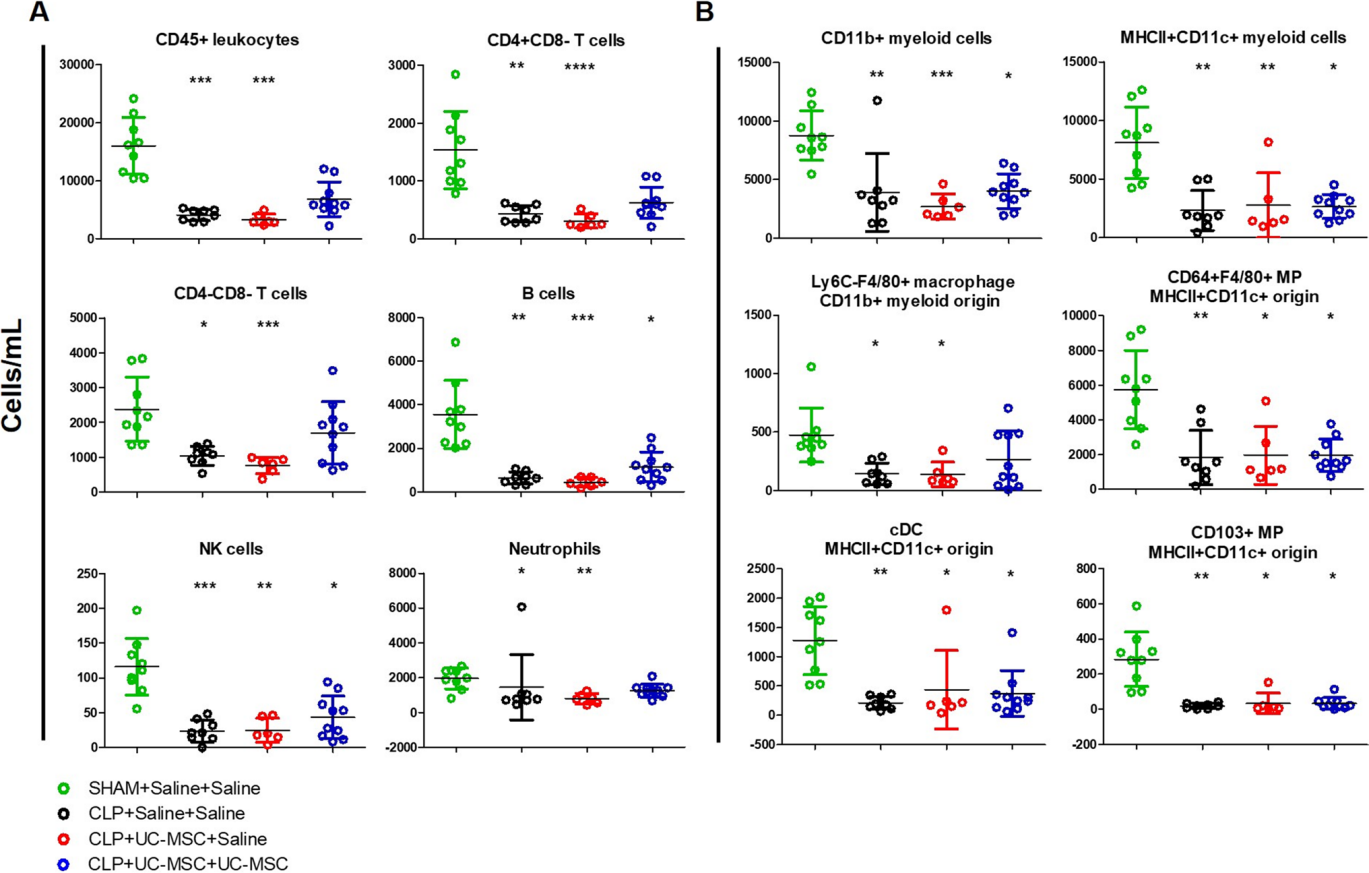
*Flow cytometric analysis of intra-renal immune cell subpopulations at 48 h following Sham or CLP procedures with saline, single-dose hUC-MSC and double-dose hUC-MSC:* Total cell numbers of intra-renal **a** lymphoid and **b** myeloid cell subpopulations quantified by multi-colour flow cytometry of CD45-enriched cell suspensions from collagenase/DNase-digested kidneys prepared at 48 h post-procedure from four groups of mice: SHAM+Saline+Saline (*n* = 9), CLP + Saline+Saline (*n* = 8), CLP + UC-MSC + Saline (*n* = 6) and CLP + UC-MSC + UC-MSC (*n* = 10). Statistical analysis: Non-Gaussian distribution: Kruskal-Wallis test with Dunn’s multiple comparison test, p < 0.05. Gaussian distribution: One-way ANOVA with Bonferroni multiple comparison post-test and with 95% confidence interval. *Significantly different from SHAM group (*p < 0.05, **p < 0.01, ***p < 0.001). Abbreviations: MP = mononuclear phagocytes

In kidney cell suspensions from the same experimental groups, magnetic enrichment and multi-colour flow cytometry were used to quantify total (CD45^+^) intra-renal immune cells as well as several myeloid and lymphoid immune cell subpopulations (Fig. 5). As shown, kidneys of the surviving animals from the untreated and single-dose hUC-MSC CLP groups demonstrated reduced numbers of total immune cells and of all myeloid and lymphoid subpopulations compared to the sham controls. For the double-dose hUC-MSC-group, however, intra-renal numbers of total immune cells as well as of CD4^+^ T-cells, CD4^−^/CD8^−^ (double negative) T-cells, neutrophils and Ly6C^−^/F4/80^+^ macrophages did not differ significantly from those of sham controls. These observations were consistent with sepsis-associated depletion of intra-renal myeloid and lymphoid immune cell populations at the time-point studied that was partially reversed by double- but not single-dose i.v. hUC-MSC. Analysis of dead/dying cells among the renal CD45^+^ cells for each group (based on positive staining for the viability dye eFluor 506) revealed reduced proportions of live cells in samples from untreated and single-dose hUC-MSC groups compared to sham controls but not in those from the double-dose group (Supplementary Fig. S2). Overall, the results for analyses focussed on the kidneys at 48 h after onset of polymicrobial sepsis, suggested that, despite the inconsistent rise in urea/creatinine and lack of overt acute tubular necrosis, there was a substantial acute injury response and increased intra-renal immune cell death. Furthermore, repeated i.v. dosing of hUC-MSC was associated with evidence of amelioration of these abnormalities.

In keeping with a sepsis-associated immune depletion that also impacted other organs and tissues, flow cytometric analysis of cell suspensions from lungs and spleens of the same animals at 48 h post-CLP also demonstrated reduced total numbers of CD45^+^ cells and of some lymphoid and myeloid subpopulations, that was less marked in the double-dose treated group (Supplementary Figs. S3 and S4).

### Cluster Analysis of Multiple Blood Analytes Reveals Response Trends and Intra-Group Heterogeneity Following Repeated Dosing of hUC-MSC in CLP

**Fig. 6:**
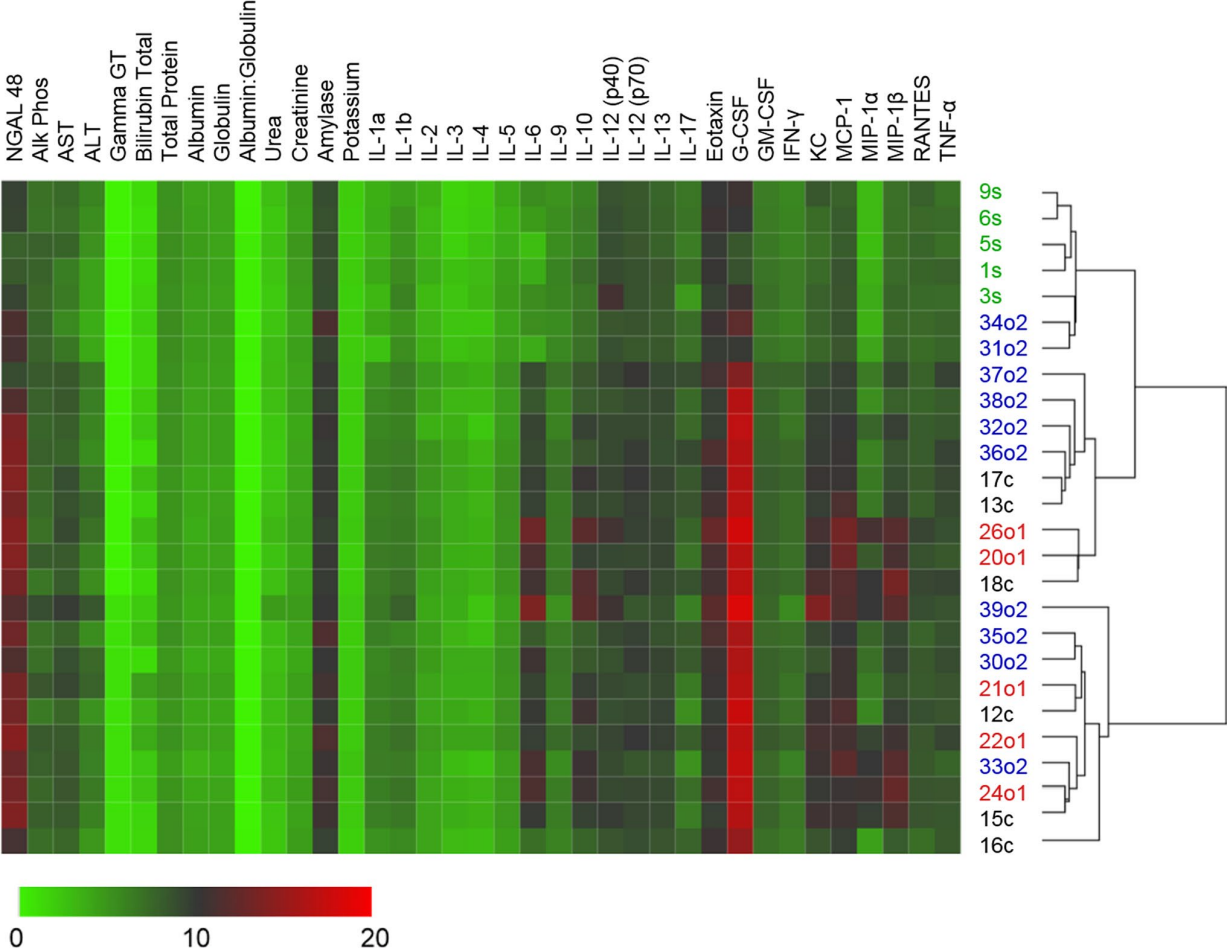
*Unsupervised hierarchical clustering of response patterns of control and hUC-MSC-treated animals:* Average linkage Euclidean distance clustering of data from Sham (green, s, *n* = 5), CLP (black, c, *n* = 6) and UC-MSC-treated animals with single (red, o1, *n* = 5) or double dose (blue, o2, *n* = 10) of 1 × 10^6^ UC-MSC. The lowest level is represented by green, while highest level is represented by red. All data except NGAL and Albumin:Globulin subjected to Log2 transformation prior to clustering

In keeping with the complex nature of polymicrobial sepsis, many of the analyses shown in Figs. 2, 3, 4 and 5 for untreated, single- and double-dose hUC-MSC-treated CLP groups were associated with high intra-group variability and with trends toward beneficial effects of repeated dosing or non-significant differences to the sham control group. To better understand whether these results represented evidence of a specific hUC-MSC-associated “responder profile” among that experimental group, an unsupervised hierarchical clustering analysis was performed of all factors quantified in blood of surviving animals at the 48-h time-point after initiation of CLP (Fig. 6). As shown, the cluster containing all 5 of the sham group animals was divided from the rest of the animals by the greatest Euclidean distance and this same cluster also included 2/10 double-dose i.v. hUC-MSC-treated CLP animals. Other clusters contained mixed populations from each of the 3 CLP groups. Overall, while suggesting that animals treated with two sequential i.v. doses of hUC-MSC were more likely than those from other groups to resemble non-septic animals, this analysis highlighted the wide variability in systemic markers of sepsis for the CLP model and did not indicate a distinct signature of disease modulation following hUC-MSC administration.

## Discussion

Sepsis and its frequent complication SA-AKI are major public health challenges due to the continued lack of effective treatments and disappointing results from late-stage clinical trials [2, 3]. Several recent pre-clinical studies have reported results indicating that MSC of various sources and their products have positive effects on disease severity and survival in models of sepsis and AKI [7, 8, 11–15]. Nonetheless, the clinical benefits of MSC in sepsis and SA-AKI remain unproven with only limited data available from human patients [5]. To date, clinical trials have documented that single-dose i.v. MSC infusion in the setting of LPS administration, sepsis and ARDS is safe and feasible, [16–19] but does not overtly reduce death from sepsis-related organ failure [17]. In this study, we compared the effects of single- and double-dose i.v. administration of a distinctive human MSC therapeutic product (CD362-selected hUC-MSC) in the mouse CLP model of polymicrobial sepsis. Initially, in keeping with recently-reported results for this same MSC product in rat models of bacterial pneumonia and sepsis [23, 24], we confirmed the potential for early administration of hUC-MSC to modulate LPS-induced systemic inflammation in mice. In a mouse CLP model of polymicrobial sepsis, however, our results indicated that single doses of hUC-MSC administered i.v. 4 h following induction of fecal peritonitis exerted no clear beneficial effect on systemic inflammation or organ-specific (renal) tissue injury. In contrast, administration of a second i.v. dose of hUC-MSC 24 h later resulted in multiple signals of ameliorated inflammation and renal injury by 48 h following onset of sepsis. Despite this, a survival benefit for the double-dose regimen was not definitively demonstrated. Our results highlight the importance of performing in vivo studies of MSC therapeutic products in multiple models and of reporting both positive and negative pre-clinical outcomes in order to better inform clinical translation and trial design. Taken together, our results also provide evidence that the limited or absent benefit of early, single-dose regimens of anti-inflammatory MSC products in sepsis trials may be at least partially overcome by repeated dosing.

In the mouse LPS model, a single, early dose of hUC-MSC modulated systemic levels of both innate and adaptive inflammatory mediators for at least 72 h, albeit without an overt effect on severity scores and survival. Phenotypic analysis of peritoneal macrophages provided evidence that hUC-MSC administration was associated with a predicted local immune modulatory effect – skewing of macrophages toward M2 polarization, which has been linked to resolution of inflammatory injury and promotion of tissue repair [29, 35] and is likely to be mediated by MSC cross-talk with resident myeloid cells [36]. Indeed, recent studies have reported that therapeutic immunomodulation by MSC in the setting of sepsis may be dependent on their phagocytosis by myeloid cells (mononuclear phagocytes) which then undergo alternative activation resulting in the production of IL-10 and other paracrine anti-inflammatory mediators [7, 27, 37]. Although it would have been of interest to determine whether the benefits of such immunomodulation are enhanced by repeated doses of hUC-MSCs following LPS administration, we reasoned that an experimental model which better reflected an evolving sepsis would have more clinical relevance. Thus, having confirmed hUC-MSC biological activity in mice using LPS administration, the mouse CLP model was used for characterization of anti-inflammatory effects related to repeated dosing and quantification of kidney-specific effects of hUC-MSC in the setting of polymicrobial sepsis and SA-AKI. In our hands, this model was associated with moderately severe sepsis (approximately 70%, 50% and 40% survival at 48, 96 and 168 h respectively in untreated animals) without overt liver and kidney failure. Our observations for the model, including mortality rates and trends in serum liver parameters and albumin, are in keeping with the very comprehensive profiling of mouse CLP reported by Li et al. [38], which also documents reduced body temperature, blood pressure and heart rate during the first 48 h post-CLP. Interestingly, while Li et al. documented increased serum creatinine and blood urea nitrogen at 8 and 16 h post-CLP, their results indicate that these renal functional biomarkers had fallen to normal (or below normal) levels by 48 h – perhaps reflecting evolving effects of altered metabolism/muscle mass on these biomarkers as the model progresses [38]. Consistent with this, analysis of serum/plasma and kidney tissue at 48 h indicated significant systemic inflammation and renal injury response without overt evidence of ischemic damage/necrosis. These latter analyses provided the clearest evidence for a modulatory effect of the double-dose hUC-MSC regimen on the severity of polymicrobial sepsis when compared to single-dose administration which, in contrast to results recently reported in a rat CLP model [24], was indistinguishable from the saline-treated CLP group across all indices examined. In particular, the clinically-relevant AKI biomarker, NGAL, proved to be a valuable discriminator of sepsis severity and treatment effect in this model and may be an important biomarker for future clinical trials of cell therapies in sepsis and SA-AKI [33, 39].

Our results for the effect of i.v. MSC on survival in small animal models of sepsis are in contrast to some other reports [28, 40] but, notably, are in keeping with observed effects of allogeneic MSCs in human clinical trials [5, 16–18, 41]. In this regard, we would highlight the potential role of publication bias – specifically, selective publication of results reflecting positive effects – as an important driver of unrealistic expectations for the efficacy of single-dose MSC regimens and other advanced therapies in the earliest stages of clinical translation [42]. Indeed, Sun et al., in a recent meta-analysis of 29 animal studies of the efficacy of MSC therapies in sepsis, detected significant publication bias and lack of clarity in regard to optimal cell dose among these pre-clinical reports [43]. Nevertheless, we also report distinctive positive findings that may help to advance translational goals for UC-MSCs in sepsis or other systemic and organ-specific inflammatory diseases, such as AKI, liver or respiratory diseases. In both models, these molecular changes indicate complex interactions of hUC-MSC with the Th1 and Th2 immune response. Such effects on T-effector cell activation and T helper phenotype balance may play a key role in modulation of the acute phase of sepsis as indiscriminate, dysregulated activation of immune effectors resulting in high levels of circulating cytokines contribute to multi-organ failure [44] and, in the case of T-cells, may be followed by widespread apoptosis and subsequent immune deficiencies [45]. It has also been shown that MSC administration may decrease localized tissue inflammation by regulating cytokine homeostasis and decreasing the traffic of immune cells into organs [44]. In keeping with this, our quantitative analysis of a range of intra-renal immune cell populations 48 h following CLP-indued sepsis revealed sepsis-induced deficiencies affecting both innate and adaptive effectors, including loss of double-negative T cells which have been recently reported to be early responders to AKI [46]. Notably, for double- but not single-dose hUC-MSC-treated animals, there was evidence of reversion of intra-renal immune cell depletion. As similar trends were also observed in lungs and spleen, our results indicate the potential for repeated i.v. dosing of MSC to broadly ameliorate immune cell depletion from non-lymphoid and lymphoid organs – a facet of sepsis that has been linked to subsequent mortality due to secondary infection [47, 48]. Based on the additional observation of increased cell death among intra-renal CD45^+^ cells in untreated CLP animals, it is plausible that this reflects direct or indirect effects of hUC-MSC to reduce mitochondrial dysfunction and pro-apoptotic signalling[49–51]. Nonetheless, more focussed experiments will be required to fully elucidate the mechanisms by which systemic MSC administration preserves myeloid and/or lymphoid cell numbers in sepsis and to determine whether they can be exploited therapeutically.

Given the inherent variability we and others observe with individual outcomes for the mouse CLP model, we hypothesized that hierarchical clustering analysis of quantitative readouts for a range of circulating inflammation-related mediators at 48 h would help to better define a distinctive “responder signature” among MSC-treated animals. Although 60% of the double-dose group clustered in a pattern that separated them from the majority of the single dose- and untreated groups and was closer to the sham group, no very clear multi-analyte profile of disease modulation could be identified. A principal component analysis (PCA) approach yielded a similar conclusion (data not shown). It should be acknowledged that data from animals that failed to survive to 48 h could not be acquired and it is possible that analyses at one or more earlier time-points could provide a more distinctive responder/non-responder separation. Nonetheless, this analysis highlights the complexity of inter-individual variation that is inherent to animal models of sepsis and cell therapies even with close attention to principles of good experimental design [52, 53] and that reflects similar challenges faced in the clinical application of novel therapies to sepsis [18, 20, 41, 54, 55].

Some limitations of the study should be acknowledged. In the first place, we have focussed on investigating the in vivo effects of CD362-selected hUC-MSC as this cell product is undergoing clinical trial for other inflammation-driven diseases including COVID-19-associated ARDS. This study design precluded gaining further insight into the comparative effects, in sepsis and SA-AKI, of the cell product tested with those of unselected hUC-MSC or with MSC derived from bone marrow or other tissues. Secondly, as the cell doses used were chosen on the basis of prior studies of human MSC anti-inflammatory effects in mice, it is not possible to determine whether multiple administrations of higher or lower cell numbers provides greater benefit. Finally, as the group sizes for the CLP model experiments were powered to address hUC-MSC effects on a systemic inflammatory biomarker of sepsis/SA-AKI severity, it is possible that larger group sizes would have more clearly defined the effect of repeated doses on survival. While these issues further emphasize the need for sequential pre-clinical experiments that adhere as closely as possible to the key parameters required for optimal clinical trial design, the results we present here support a continued focus on multi-dose regimens of anti-inflammatory MSC in sepsis and SA-AKI.

## Supplementary Information

ESM 1 (DOCX 21 kb)

ESM 2 (DOCX 2405 kb)

## Data Availability

The data that support the findings of this study are available from the corresponding author upon reasonable request.

## Abbreviations

MSC: Mesenchymal stromal cells
SA-AKI: Sepsis-associated acute kidney injury
hUC-MSC: Human umbilical cord-derived MSC
LPS: Lipopolysaccharide
CLP: Cecal ligation and puncture
Intravenous: i.v.
Intraperitoneal: i.p.
NGAL: neutrophil gelatinase-associated lipocalin
