# Supplementary material for: Comparison of Single and Repeated Dosing of Anti-Inflammatory Human Umbilical Cord Mesenchymal Stromal Cells in a Mouse Model of Polymicrobial Sepsis
Source: Stem Cell Rev Rep. 2022 Jan 10;18(4):1444–60. doi: 10.1007/s12015-021-10323-7 (PMC8747454; doi:10.1007/s12015-021-10323-7)
Supplement: Supplementary file 1 — (DOCX 21 kb) [file 12015_2021_10323_MOESM1_ESM.docx]

**Comparison of single and repeated dosing of anti-inflammatory human umbilical cord mesenchymal stromal cells in a mouse model of polymicrobial sepsis**

Barbara Fazekas, Senthilkumar Alagesan, Luke Watson, Olivia Ng, Callum M. Conroy, Cristina Català, Maria Velasco de Andres, Neema Negi, Jared Q. Gerlach, Sean O Hynes, Francisco Lozano, Stephen J. Elliman and Matthew D. Griffin

**Supplementary Material**

**Supplementary Methods**

*Flow cytometric analysis of kidney, lung and spleen immune cell subpopulations:* The spleen samples were mashed through a 40µM filter in Hank’s balanced salt solution (HBSS, Sigma-Aldrich). The kidneys were de-capsulated prior to digestion. Decapsulated kidneys and freshly-dissected lungs were manually chopped and digested in 1mL of Dulbecco’s modified Eagle’s medium (DMEM) or HBSS medium containing 0.25 or 0.20unit/mL of recombinant collagenase class I and II originating from *Clostridium histolyticum* (KITECH, South Korea), 0.06 or 0.05mg/mL or of Thermolysine (Sigma-Aldrich, Arklow, Ireland) and 0.4 or 0.2mg/mL or 0.2mg/mL DNase I (Sigma-Aldrich), respectively. Samples were incubated at 37°C for 40 minutes with rotation at 250 rpm. Upon completion of digestion, cell suspensions were washed with HBSS (Sigma-Aldrich), strained through a 40μm cell strainer and treated with ACK lysis buffer (150mM NH_4_Cl, 10mM KHCO_3_ and 0.1mM Na_2_EDTA dissolved in dH_2_O, pH 7.2-7.4). The suspensions were washed once in HBSS, then twice with Dulbecco’s phosphate buffered saline (D-PBS, Thermo Fisher Scientific) and then suspended in FACS buffer at 2.5x10^5^ cells/mL. For CD45^+^ cell enrichment of kidney cell suspensions, magnetic column separation of kidney cell suspensions was carried out in MACS buffer (PBS, 0.5% BSA and 100mM EDTA) by manufacturer-recommended protocols using MS columns and an OctoMACS® separator (Miltenyi Biotec Inc., Bisley, Surray, UK). Aliquots of the final kidney, lung and spleen cell suspensions were stained with combinations of fluorochrome-labelled antibodies for 30 min at 4°C: anti-TCRβ-PE (H57-597), anti-CD19-FITC (1D3) and anti-CD45-FITC (EM-05) from Immunotools, Friesoythe, Germany; anti-CD4-PerCp-Cy5.5 (RM4-5), anti-CD11c-PE (HL3) and anti-CD45-V450 (30F11) from BD Biosciences, Berkshire, UK; anti-CD8 -PE-Cy7 (53-6.7), anti-NK1.1-APC (DX5), anti-CD45-APC-Cy7 (30-F11), anti-Ly6C-PerCp-Cy5.5 (HK1.4), anti-Ly6G (1A8), anti-CD206-V450 (C068C2) and anti-MHC-II-FITC (AF6-120.1) from Biolegend, London, UK; anti-F4/80 (BM8) from Invitrogen/eBioSciences (Bio-Sciences Ltd., Dublin, Ireland); and anti-CD64-APC-Cy7 (REA286) from Miltenyi Biotech. Cell viability was analysed by using the fixable Viability Dye eFluor™ 506 (Invitrogen/eBioSciences). After staining, the cells were washed in FACS buffer, fixed in 1% paraformaldehyde and re-suspended in FACS buffer before being analyzed on a FACS Canto II cytometer (BD Biosciences). A total of 150,000 events were collected for each sample and the acquired data were analyzed using FlowJo 6 software. Fluorescence minus one (FMO) controls were prepared for fluorophores identifying infrequent cell populations and were used to define positive staining. Compensation was performed with OneComp beads (eBioscience) stained with appropriate antibodies. Absolute cell numbers were calculated by using the CountBright Absolute Counting Beads (Thermo Fisher Scientific, Bio-Sciences Ltd.).

*ELISA of plasma for NGAL:* 96-well microtiter plates (Thermo-Fisher Scientific) were coated with the capture antibody, and non-specific binding sites were blocked with reagent diluent (1% BSA in PBS, pH 7.2–7.4). Adequately-diluted plasma samples (0.4x10^3^, 10^4^ to 10^5^-fold) were incubated in duplicates for 2 hours, and then the detection antibody was added. Next, Streptavidin-HRP was added for 20 minutes, followed by addition of 50μL of 3,3',5,5'-Tetramethylbenzidine (TMB) substrate (Sigma-Aldrich). Between each step, plates were washed three times with wash buffer. The enzymatic reaction was terminated by addition of 25μL of stop solution containing H_2_SO_4_. The optical density was measured with Victor3™ 1420 Multilabel Counter (PerkinElmer, Wallac Oy, Finland) at 450nm with wavelength correction set to 544nm. The NGAL concentrations were calculated with Excel software (Microsoft Office), using a four-parameter logistic curve-fit.

*Histologic staining of kidney tissue sections:* For hematoxylin and eosin (H&E) staining, sections were dewaxed in xylene, rehydrated, and stained in Gill no. 3 hematoxylin (Sigma-Aldrich) followed by eosin (Sigma-Aldrich) before being dehydrated through graded alcohols, cleared in xylene, and then covered with DPX mounting medium and coverslips. For periodic acid-Schiff (PAS) staining, sections were dewaxed in xylene, rehydrated, and then oxidized in 0.5% periodic acid solution. Sections were then washed and incubated in Schiff reagent (Sigma-Aldrich) for 20 minutes in dark. Following counterstaining in Gill no. 3 hematoxylin (Sigma-Aldrich), sections were dipped in acid alcohol and ammonia water, dehydrated, cleared in xylene, and mounted in DPX medium.

*Semi-quantitative scoring of kidney injury:* Individual fields from each stained tissue sections were scored in blinded fashion according to the following scoring system: 0 = no lesion; 1 = minimal or focal changes affecting less than 20% of the field; 2 = mild changes or the extension of the lesion to approximately 25% of the field; 3 = moderate changes or the extension of the lesion from 25% to 50% of the field; 4 = severe changes or the extension of the lesion to more than 50% of the field. The total injury score (A or B) consisted of the sum of the scores from three separate histology sections per kidney.

**Supplementary Table S1:** Flow cytometry-based surface marker staining combinations for defined immune cell sub-populations in CD45-enriched kidney cell suspensions

| **Immune Cell Type** | **Surface Staining Combination** |
| --- | --- |
| M1 Macrophage | Ly6C-CD11b+CD68+ |
| M2 Macrophage | Ly6C-CD11b+CD206+ |
| CD4 T cells | CD45^+^TCRβ^+^CD4^+^CD8^-^ |
| CD8 T cells | CD45^+^TCRβ^+^CD4^-^CD8^+^ |
| Double negative (DN) T cells | CD45^+^TCRβ^+^CD4^-^CD8^-^ |
| B cells | CD45^+^TCRβ^-^CD4^-^CD8^-^CD19^+^ |
| NK cells | CD45^+^TCRβ^-^CD4^-^CD8^-^CD19^-^NK1.1^+^ |
| Myeloid cells | CD45^+^CD11b^+^ |
| Neutrophils | CD45^+^CD11b^+^Ly6G^+^ |
| Mononuclear phagocytes | CD45^+^CD11b^+^Ly6G^-^CD11c^-^F4/80^+/-^Ly6C^+/-^ |
| CD206^+^ Macrophage-like cells | CD45^+^CD11b^+^Ly6G^-^CD11c^-^F4/80^+/-^Ly6C^+/-^CD206+ |
| Dendritic cells | CD45^+^Ly6G^-^CD11b^-^CD11c^+^ |
| CD11c^+^ Myeloid cells | CD45^+^CD11c^+^MHC II^+^ |
| Conventional (c)DC | CD45^+^(MHC II^+^CD11c^+^)(CD64^-^F4/80^-^) |
| CD103^+^ DCs | CD45^+^(MHC II^+^CD11c^+^)(CD64^-^F4/80^-^)CD103^+^ |
| CD11b ^+^ DCs | CD45^+^(MHC II^+^CD11c^+^)(CD64^-^F4/80^-^)CD11b^mid/high^ |
| Monocytes & Macrophages | CD45^+^(MHC II^+^CD11c^+^)(CD64^mid/high^F4/80^mid/high^) |
