# Supplementary material for: Comparison of Single and Repeated Dosing of Anti-Inflammatory Human Umbilical Cord Mesenchymal Stromal Cells in a Mouse Model of Polymicrobial Sepsis
Source: Stem Cell Rev Rep. 2022 Jan 10;18(4):1444–60. doi: 10.1007/s12015-021-10323-7 (PMC8747454; doi:10.1007/s12015-021-10323-7)
Supplement: Supplementary file 2 — (DOCX 2405 kb) [file 12015_2021_10323_MOESM2_ESM.docx]

**Supplementary Figure S1**

**
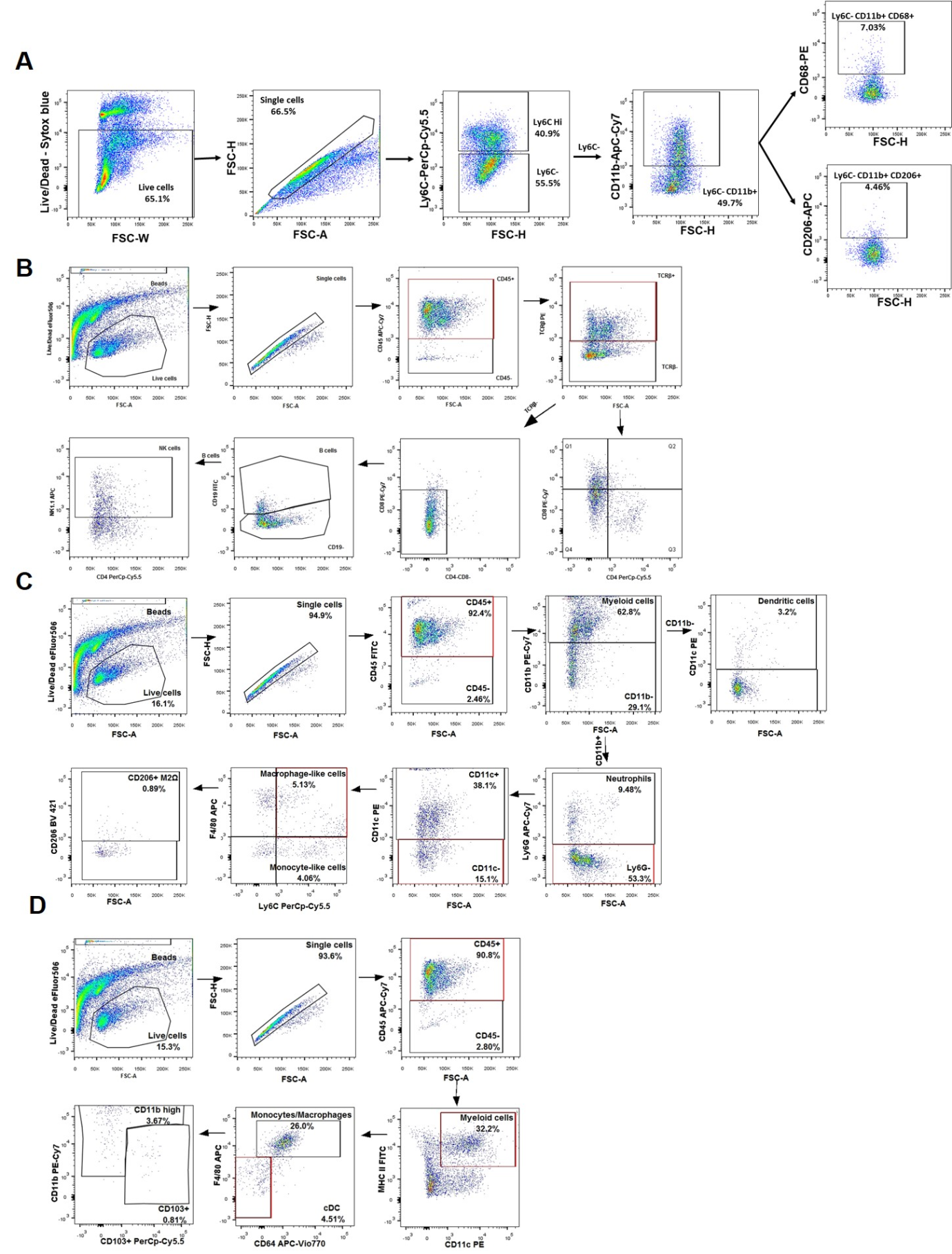
**

**Supplementary Figure S1.** *Representative examples of gating strategies for phenotypic analysis of immune cell subpopulations by flow cytometry:* **A)** Gating strategy for peritoneal macrophages. **B)** Gating strategies for renal lymphoid cell populations **C-D)** Gating strategies for renal myeloid cell populations**.**

**Supplementary Figure S2**

**
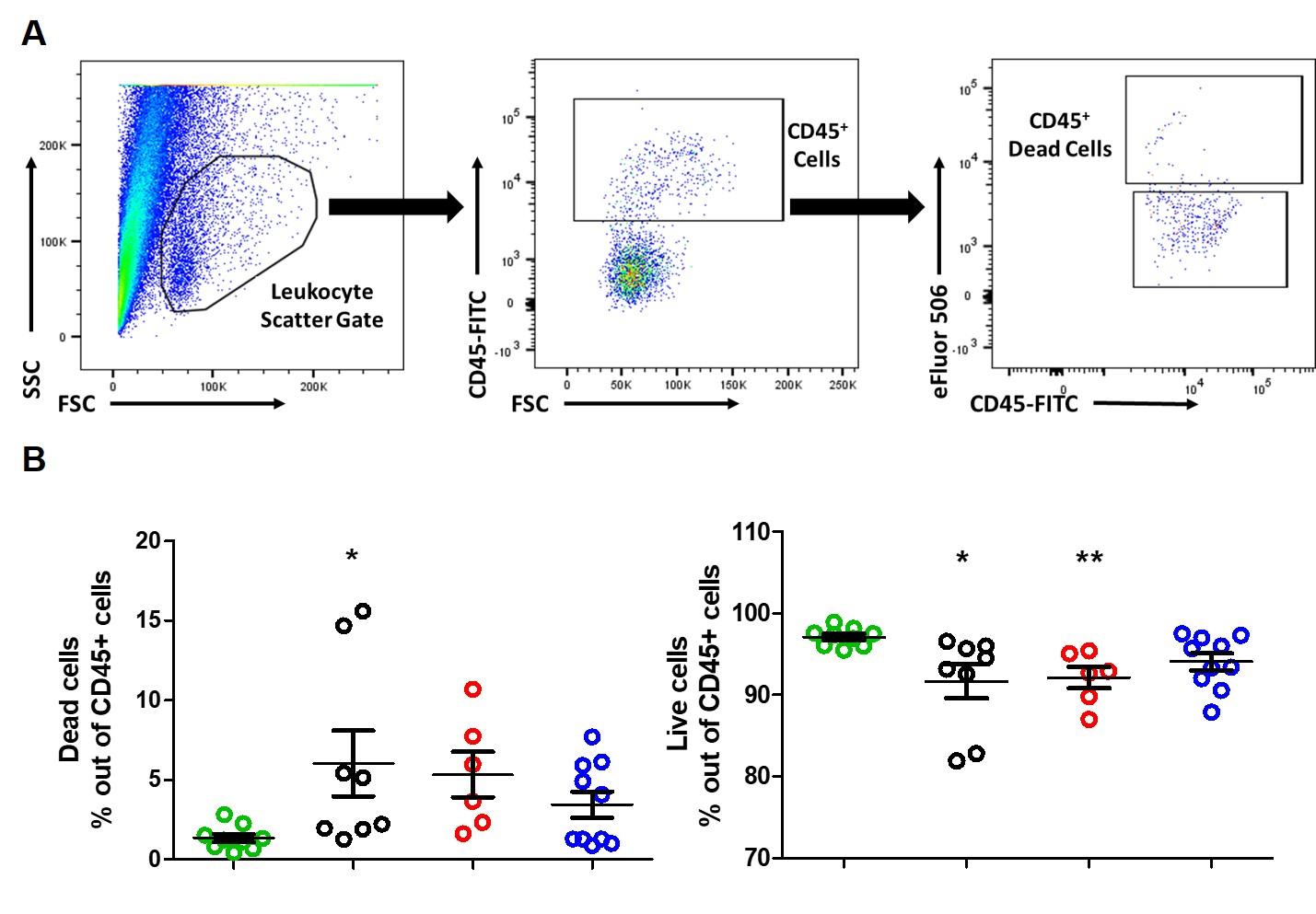
**

**Supplementary Figure S2.** *Flow cytometric analysis of live and dead CD45+ immune cells in kidneys at 48 hours following Sham or CLP procedures with saline, single-dose hUC-MSC and double-dose hUC-MSC:*  **A)** A representative example of the gating strategy used to subdivide kidney CD45^+^ cells into dead (eFlour506^+^) and live (eFlour506^-^) cells is shown. **B)** Graphical representations of the proportions of dead cells (left) and live cells (right) among the total CD45^+^ kidney cells quantified by multi-colour flow cytometry of cell suspensions prepared at 48 hours post-procedure from four groups of mice: SHAM+Saline+Saline (green symbols, n=9), CLP+Saline+Saline (black symbols, n=8), CLP+UC-MSC+Saline (red symbols, n=6) and CLP+UC-MSC+UC-MSC (blue symbols, n=10). Statistical analysis: Non-Gaussian distribution: Non-parametric Kruskal-Wallis test with Dunn’s multiple comparison test, p<0.05. Gaussian distribution: One-way ANOVA with Bonferroni multiple comparison post-test and with 95% confidence interval. *Significantly different from SHAM group (*p<0.05, **p<0.01).

**Supplementary Figure S3**

**
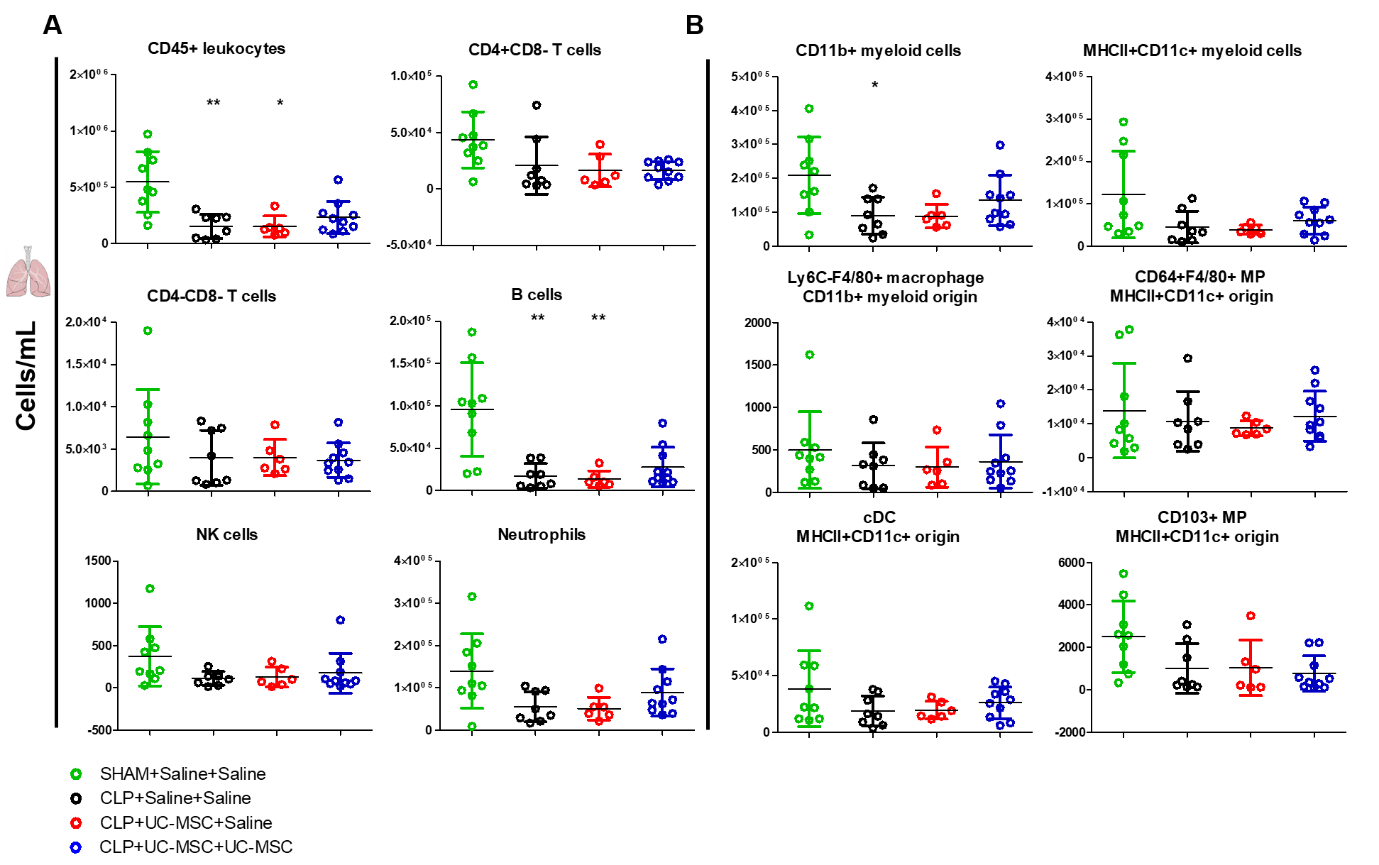
**

**Supplementary Figure S3.** *Flow cytometric analysis of immune cell subpopulations in lungs at 48 hours following Sham or CLP procedures with saline, single-dose hUC-MSC and double-dose hUC-MSC:* Total lung cell numbers of **A)** all immune cells (CD45^+^ leukocytes) and of individual lymphoid and **B)** myeloid cell subpopulations quantified by multi-colour flow cytometry of cell suspensions prepared at 48 hours post-procedure from four groups of mice: SHAM+Saline+Saline (n=9), CLP+Saline+Saline (n=8), CLP+UC-MSC+Saline (n=6) and CLP+UC-MSC+UC-MSC (n=10). Statistical analysis: Non-Gaussian distribution: Non-parametric Kruskal-Wallis test with Dunn’s multiple comparison test, p<0.05. Gaussian distribution: One-way ANOVA with Bonferroni multiple comparison post-test and with 95% confidence interval. *Significantly different from SHAM group (*p<0.05, **p<0.01). Abbreviations: MP=mononuclear phagocytes.

**Supplementary Figure S4**

**
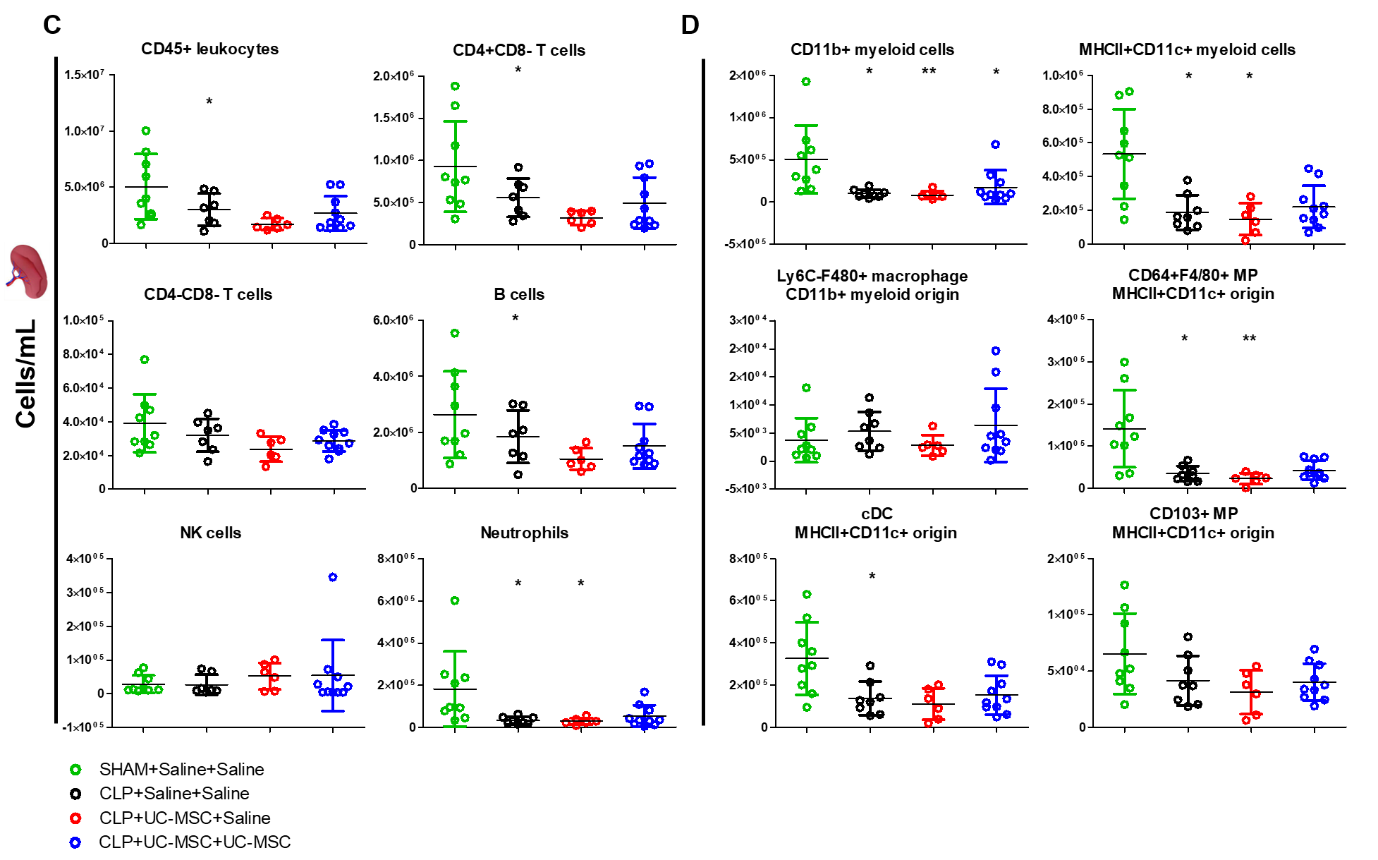
**

**Supplementary Figure S4.** *Flow cytometric analysis of immune cell subpopulations in spleen at 48 hours following Sham or CLP procedures with saline, single-dose hUC-MSC and double-dose hUC-MSC:* Total spleen cell numbers of **C)** all immune cells (CD45^+^ leukocytes) and of individual lymphoid and **D)** myeloid cell subpopulations quantified by multi-colour flow cytometry of cell suspensions prepared at 48 hours post-procedure from four groups of mice: SHAM+Saline+Saline (n=9), CLP+Saline+Saline (n=8), CLP+UC-MSC+Saline (n=6) and CLP+UC-MSC+UC-MSC (n=10). Statistical analysis: Non-Gaussian distribution: Non-parametric Kruskal-Wallis test with Dunn’s multiple comparison test, p<0.05. Gaussian distribution: One-way ANOVA with Bonferroni multiple comparison post-test and with 95% confidence interval. *Significantly different from SHAM group (*p<0.05, **p<0.01). Abbreviations: MP=mononuclear phagocytes.
